# The relationship between endorsing reporting guidelines or trial registration and the impact factor or total citations in surgical journals

**DOI:** 10.7717/peerj.12837

**Published:** 2022-01-25

**Authors:** Jing Zhou, Jianqiang Li, Jingao Zhang, Bo Geng, Yao Chen, Xiaobin Zhou

**Affiliations:** 1 Department of Epidemiology and Health Statistics, Qingdao University, Qingdao, Shandong, China; 2 Editorial Office of Journal of Precision Medicine, Qingdao University, Qingdao, Shandong, China

**Keywords:** Reporting guidelines, Trial registration, Surgery, Journal impact factor, Total cites

## Abstract

**Background:**

A journal’s impact factor (IF) and total citations are often used as indicators of its publication quality. Furthermore, journals that require authors to abide by reporting guidelines or conduct trial registration generally have a higher quality of reporting. In this study, we sought to explore the potential associations between the enforcement of reporting guidelines or trial registration and a surgical journal’s IF or total citations in order to find new approaches and ideas to improve journal publication quality.

**Methods:**

We examined surgical journals from the 2018 Journal Citation Report’s Expanded Scientific Citation Index to quantify the use of reporting guidelines or study registration. We reviewed the “instructions for authors” from each journal and used multivariable linear regression analysis to determine which guidelines were associated with the journal IF and total citations. The dependent variable was the logarithm base 10 of the IF in 2018 or the logarithm base 10 of total citations in 2018 (the results were presented as geometric means, specifically the ratio of the “endorsed group” results to “not endorsed group” results). The independent variable was one of the requirements (endorsed and not endorsed). Models adjust for the publication region, language, start year, publisher and journal size (only used to adjust total citations).

**Results:**

We included 188 surgical journals in our study. The results of multivariable linear regression analysis showed that journal IF was associated (*P* < 0.01) with the following requirements: randomized controlled trial (RCT) registration (geometric means ratio (GR) = 1.422, 95% CI [1.197–1.694]), Consolidated Standards of Reporting Trials (CONSORT) statement (1.318, [1.104–1.578]), Preferred Reporting Items for Systematic Reviews Meta-Analyses (PRISMA) statement (1.390, [1.148–1.683]), Strengthening the Reporting of Observational Studies in Epidemiology (STROBE) statement (1.556, [1.262–1.919]), Standards for Reporting Diagnostic Accuracy (STARD) statement (1.585, [1.216–2.070]), and Meta-analysis of Observational Studies in Epidemiology (MOOSE) statement (2.113, [1.422–3.133]). We found associations between the endorsement of RCT registration (GR = 1.652, 95% CI [1.268–2.153]), CONSORT (1.570, [1.199–2.061]), PRISMA (1.698, [1.271–2.270]), STROBE (2.023, [1.476–2.773]), STARD (2.173, [1.452–3.243]), and MOOSE statements (2.249, [1.219–4.150]) and the number of total citations.

**Conclusion:**

The presence of reporting guidelines and trial registration was associated with higher IF or more total citations in surgical journals. If more surgical journals incorporate these policies into their submission requirements, this may improve publication quality, thus increasing their IF and total citations.

## Introduction

For research institutions, universities, and individual scholars to understand a publication’s impact, the use of bibliometric indices is crucial (Roldan-Valadez et al., 2019). There are many bibliometric tools used to measure journal quality including impact factor (IF), total citations, eigenfactor score, h-index, and source normalized impact per paper (SNIP). These tools correspond to different aspects of journal performance, such as the impact, output, and reputation (Jones, Huggett & Kamalski, 2011). The IF and total citations are the two indicators considered most important to authors, medical editors, funding agencies, and the journal itself (Jones, Huggett & Kamalski, 2011; Roldan-Valadez et al., 2019).

Garfield (1996) from the Institute of Scientific Information (ISI) first proposed using reference counting to measure a publication’s impact. IF was first used for the 1961 Science Citation Index (SCI) in 1963 and has since been widely regarded as one of the primary indicators for evaluating the quality, importance, and impact of medical journals in their respective disciplines (Garfield, 1996; Roldan-Valadez et al., 2019). A given journal’s IF is calculated by dividing the number of citations the journal received in one year for articles published the previous 2 years (numerator) by the number of articles published over the previous 2 years (denominator) (Kumar, Upadhyay & Medhi, 2009). For example, Journal X’s IF for 2018 (“IF2018”) would be the total number of times the articles published in Journal X were cited in 2016 and 2017 divided by the total number of articles published by Journal X in 2016 and 2017. Journal size is a factor when determining journal subscriptions, and studies have shown that journal size is associated with longitudinal journal IF stability (Koelblinger et al., 2019). A journal may request that authors include references from previous publications in order to manipulate and increase its IF through self-citations (Ioannidis & Thombs, 2019). Even so, a journal’s IF is frequently cited in the scientific world when determining its quality and scientific output (Kumar, Upadhyay & Medhi, 2009). A journal’s total citations refer to the total number of times it has been cited by all journals in the journal citation report (JCR) database in a year, and is used to reflect the journal’s value, role, and status in the scientific community.

In seeking to improve their journals’ competitiveness and publication quality, scientific journal publishers rely on peer review (Wierzbinski-Cross, 2017), statistical review (Dexter & Shafer, 2017), and editorial policy. Editorial policy is known to affect the quality of a journal’s articles and usually includes requirements such as the disclosure of conflicts of interest (COI), copyright issues, article layout, chart format, reporting guidelines, trial registration, and data availability.

Reporting guidelines may improve reporting quality by promoting openness and transparency of research information, as well as controlling selective reporting (Sims et al., 2018). Reporting guidelines specify in detail how to standardize and comprehensively report each part of a study from the abstract to conclusion, especially in cases where a bias may be present. The guidelines typically dictate the use of checklists, flow diagrams, or explicit text (Simera et al., 2010). Certain reporting guidelines are already well known by researchers, *e.g*., the Consolidated Standards of Reporting Trials (CONSORT) statement published in 1996 (Junker et al., 1996), the Standards for Reporting Diagnostic accuracy (STARD) statement published in 2003 (Bossuyt & Reitsma, 2003), the Strengthening the Reporting of Observational Studies in Epidemiology (STROBE) statement published in 2007 (von Elm et al., 2007), and the Preferred Reporting Items for Systematic reviews and Meta-Analyses (PRISMA) statement published in 2009 (Moher et al., 2009). Experts have systematically developed reporting guidelines applicable to different types of research, added suggestions to improve scientific writing, and developed instructions for authors in specific journals (Moher et al., 2010). The Enhancing the Quality and Transparency of Health Research (EQUATOR) network was founded in 2006 and put into practice in 2008. Their website includes comprehensive reporting guidelines, and they seek to raise awareness for and promote the adoption of good publishing practices (Altman et al., 2008; Moher et al., 2008; Simera et al., 2010). The International Committee of Medical Journal Editors (ICMJE) also have recommendations for the conduct, reporting, editing, and publication of scholarly work in medical journals that are widely regarded as an effective solution to standardizing manuscript preparation and formatting (Matheson, 2011). These recommendations cover ethical issues, publication problems, and the preparation, structure, and submission of manuscripts (http://www.icmje.org/).

Clinical trial registration is also used to improve the reporting quality of articles (De Angelis et al., 2004). Trial registration promotes transparency and accountability and may also limit bias (Sims et al., 2018). Researchers and journal editors tend to publish positive rather than negative trials, which leads to the selective reporting of experimental results (Bonati & Pandolfini, 2006; Pansieri, Pandolfini & Bonati, 2015). The full implementation of clinical trial registration enables each trial to be publicly recorded, which is of great benefit to those who want to obtain comprehensive clinical evidence (McFadden et al., 2015). Section 801 of the Food and Drug Administration Amendments Act (FDAAA 801) requires that relevant clinical trials be registered within 21 days of participant enrollment. The ICMJE and some journals require clinical trial registration prior to participant enrollment (Sim et al., 2006).

Currently, most medical professionals access cutting-edge knowledge and the subject dynamics of their profession *via* medical journals. Additionally, surgeons’ clinical treatment decisions depend to a large extent on the reported results of clinical trials. Therefore, articles in surgical journals have stricter requirements. Empirical studies have shown that journals that require authors to abide by reporting guidelines and conduct trial registration generally have higher quality reporting (Smith et al., 2015; Toews et al., 2017), and these policies are effective at achieving research repeatability and improving reporting transparency (Kim et al., 2012; Simera et al., 2010).

Therefore, we conducted a systematic review of some disciplines related to clinical medicine (including internal medicine, oncology, nursing, obstetrics and gynecology, and anesthesiology), and collected the requirements for various policies and the information related to the journal for research and analysis. We found that a large number of journals have not adopted reporting guidelines or study registration, which suggests that these measures have not been fully utilized. Since journals that implement these policies have a higher quality of literature, are these policies related to publication quality and journal reputation? How is this association reflected in the journal’s IF and total citations? To investigate these questions, we took surgical journals as an example and explored the association between the presence of reporting guidelines or study registration with the IF or total citations. We hypothesized that the implementation of these measures was associated with higher IF and more total citations in surgical journals. If these policies gain the attention of surgical journals, they can provide possible new approaches and ideas for improving publication quality and journal reputation.

## Materials and Methods

### Study design

We used cross-sectional studies to investigate surgical journals’ compliance with reporting guidelines or study registration. At the same time, we extracted journal characteristic information in order to carry out a follow-up analysis. The STROBE guidelines were observed during the design, performance, analysis, and reporting sections of our study.

### Reporting guidelines or study registration

Data were collected from the sample journals with regard to adopting reporting guidelines or study registration. All requirements were sorted into the “endorsed group” or the “not endorsed group”, and we used these groupings to explore which requirements were associated with the IF and total citations. We determined whether compliance with various requirements was “required”, “recommended”, or “not mentioned”. The representative words for “recommended” compliance were “refer to”, “encourage”, or “suggest”. For “required” compliance, the representative words were “should”, “must”, or “otherwise the manuscript will not be considered for publication”. We classified “recommended” and “required” compliance as an endorsement. If these requirements were not mentioned, then this was considered not an endorsement.

### Journal characteristics

We collected some characteristic information about sample journals, including IF in 2018, total citations in 2018, publication region, publisher, start year, language, and journal size (all citable items of the journal found in the 2018 JCR). Among these, we artificially divided the languages into two groups: English and non-English (which included French, German, Spanish, and Turkish).

### Data collection

**Figure 1:**
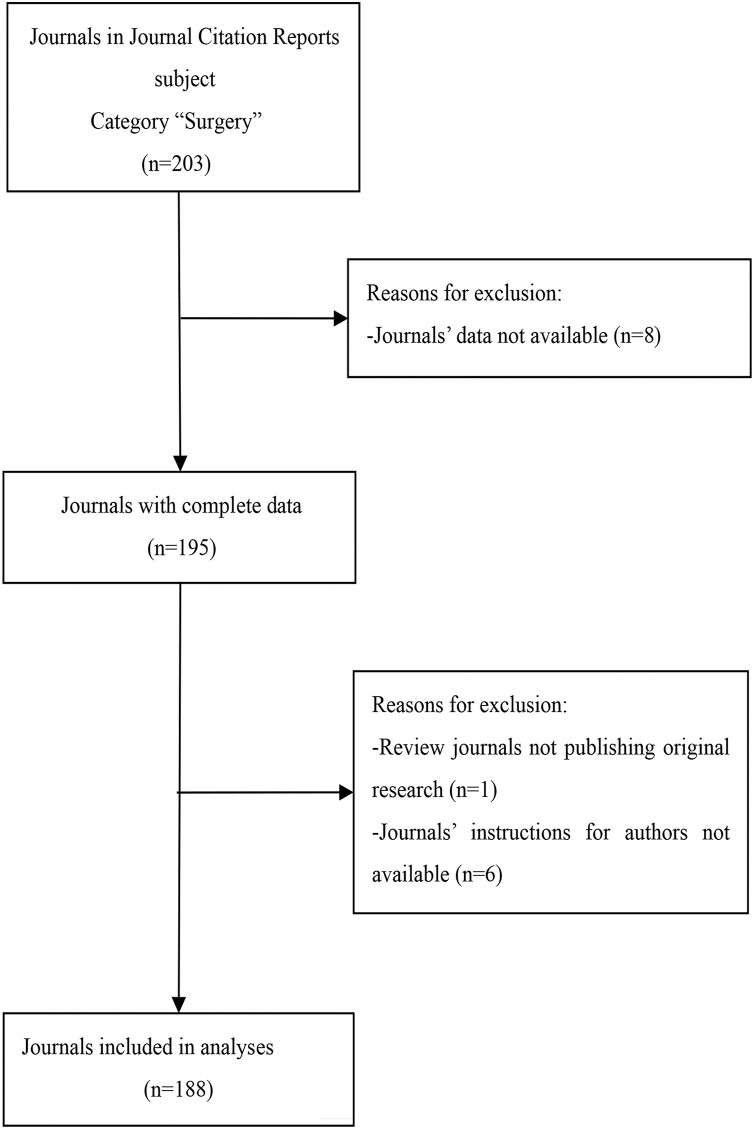
Flow diagram of included journals and reasons for exclusion.

We selected surgical journals from the 2018 JCR’s Expanded Scientific Citation Index. We excluded journals with incomplete data, those that did not publish original research, and journals whose author instructions could not be accessed. Our search resulted in a total of 188 surgical journals (Fig. 1). We collected data from March to June 2020 by evaluating each journal’s instructions for authors and related information, including author guides and guidelines, information to contributors, submission guidelines, COI information, journal policies, manuscript guidelines, publisher policies, instructions for manuscript preparation, information for authors, and submission policies. At the beginning of the study, two authors (JZ and JGZ) independently extracted data for analysis. Any discrepancies in the results were evaluated by a third author (XBZ) who rendered a final decision after discussion with all authors. Raw measurements obtained from the journals are shown in Data S1. We listed the information of journals that were excluded from this study in Data S2.

### Model hypothesis

**Figure 2:**
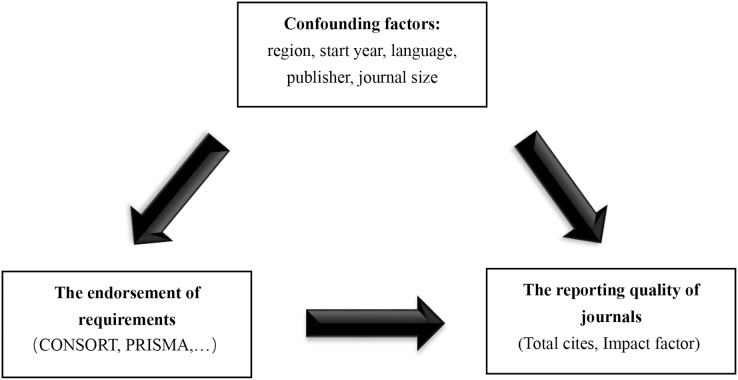
Model hypothesis diagram. CONSORT, Consolidated Standards of Reporting Trials; PRISMA, Preferred Reporting Items for Systematic Reviews and Meta-Analyses.

We hypothesized that the implementation of these measures was associated with higher IF or more total citations in surgical journals. The residual analysis results showed that the data were not subject to normality. Therefore, we performed logarithmic transformation of the dependent variable for subsequent multivariable linear regression analysis. The dependent variable was the logarithm base 10 of the IF in 2018 or the logarithm base 10 of total citations in 2018 (the results were converted back to the original scale and presented as geometric means, specifically the ratio of the results of the “endorsed group” to that of the “not endorsed group”). The independent variable was one of the requirements (endorsed and not endorsed) and the adjusted covariables were publication region, language, start year, publisher, and journal size (only used to adjust total citations). Among these, start year and journal size were continuous variables, and the rest of the covariables were categorical variables. The model hypothesis is shown in Fig. 2.

### Data analysis

Categorical variables were presented as frequencies. Based on the results of the P-P plot (or histogram), the data were not subject to normal distribution. Therefore, continuous variables were presented as median and quartile (P_25_–P_75_). After univariable linear regression analysis, we also performed multivariable linear regression analysis to adjust for the possible confounding of the factors (publication region, language, start year, publisher, and journal size) and assess whether the use of reporting guidelines and study registration was associated with the IF and total citations. We used residual analysis to diagnose the model and variance inflation factor (VIF) to judge the collinearity between independent variables. All statistical analyses were performed using SPSS Statistics 18.0 (IBM Corporation, Armonk, NY, USA). All reported *P* values were two-sided, and *P* values ≤ 0.05 were statistically significant.

## Results

### Descriptive characteristics

A total of 188 of the 203 surgical journals met the inclusion criteria and were included in our study (Fig. 1). Each journal had a website from which we obtained information for our research. The distribution of data with regards to the journal characteristics is shown in Table 1. English language journals accounted for 92.6% (*n* = 174) of the journals and 51.1% (*n* = 96) of journal editorial offices were located in North America. The median IF was 1.909 (P_25_–P_75_: 1.159–2.998). The median total citations was 3,124 (P_25_–P_75_: 1,131–7,849). The specific distribution of the IF and total citations in surgical journals is shown in Figs. S1 and S2.

**Table 1:** Characteristics of included journals (*n* = 188).

Journal characteristics	Number of journals (%) or median (P_25_–P_75_)^a^
**Geographical region of publication**	
North America	96 (51.1)
Europe without UK	49 (26.1)
UK	29 (15.4)
Others^b^	14 (7.4)
**Language**	
English	174 (92.6)
Not English^c^	14 (7.4)
**Publisher**	
Elsevier	44 (23.4)
Springer	24 (12.8)
Lippincott Williams & Wilkins	17 (9.0)
Wiley	16 (8.5)
Others^d^	87 (46.3)
**Start year**	1,989 (1,969–1,999)
**Impact Factor 2018**	1.909 (1.159–2.998)
**Total Cites 2018**	3,124 (1,131–7,849)
**Journal size** ^e^	121 (66–238)

**Notes:**

a P_25_–P_75_: The 25th percentile to 75th percentile.

b Others: including Asia and Africa.

c Not English: including French, German, Spanish and Turkish.

d Others: The minority percentage of the publishers.

e Journal size: Citable items of journal in JCR 2018.

### Endorsement of requirements

**Figure 3:**
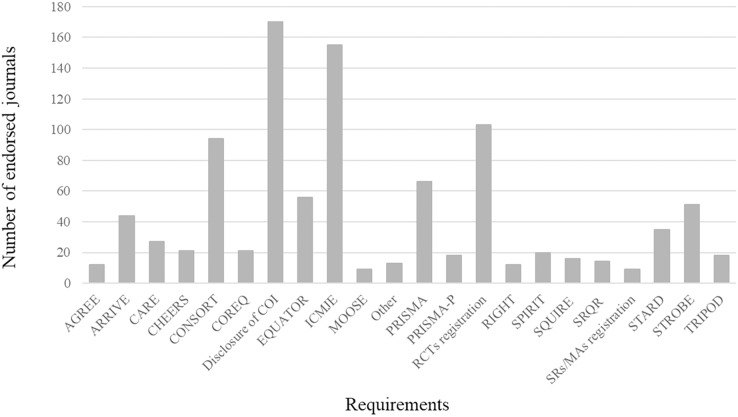
Frequency of endorsement of each requirement in included surgery journals (*n* = 188). AGREE, Appraisal of Guidelines for Research and Evaluation; ARRIVE, Animal Research: Reporting of *In Vivo* Experiments; CARE, CAse REports; CHEERS, Consolidated Health Economic Evaluation Reporting Standards; CONSORT, Consolidated Standards of Reporting Trials; COREQ, Consolidated criteria for reporting qualitative research; COI, Conflict of Interest; EQUATOR, Enhancing the Quality and Transparency Of health Research; ICMJE, International Committee of Medical Journal Editors; MOOSE, Meta-Analysis Of Observational Studies in Epidemiology; Other, The minority percentage of reporting guidelines; PRISMA, Preferred Reporting Items for Systematic reviews and Meta-Analyses; PRISMA-P, Preferred Reporting Items for Systematic Review and Meta-Analysis Protocols; RCTs, Randomized Controlled Trials; RIGHT, Reporting Items for Practice Guidelines in Healthcare; SPIRIT, Standard Protocol Items for Reporting in Trials; SQUIRE, Standards for Quality Improvement Reporting Excellence; SRQR, Standards for Reporting Qualitative Research; SRs/MAs, Systematic Reviews/Meta Analyses; STARD, Standards for Reporting Diagnostic accuracy; STROBE, Strengthening the Reporting of Observational studies in Epidemiology; TRIPOD, Transparent reporting of a multivariable prediction model for individual prognosis or diagnosis.

A total of 21 requirements (including reporting guidelines, study registration, disclosure of COI, and EQUATOR network) were endorsed by the 188 surgical journals. The frequency of these endorsements for each requirement is shown in Fig. 3. COI disclosure (*n* = 170, 90.4%) was most likely to be adopted, followed by ICMJE recommendations (*n* = 155, 82.4%) and Randomized Controlled Trials (RCT) registration (*n* = 101, 53.7%). The CONSORT statement was the most frequently endorsed (*n* = 94, 50.0%) statement, followed by the PRISMA statement (*n* = 66, 35.1%). None of the other reporting guidelines were endorsed by more than 30% of the journals. The least-endorsed reporting guideline was the Meta-Analysis of Observational Studies in Epidemiology (MOOSE) statement (*n* = 9, 4.8%), and the Systematic Reviews/Meta Analyses (SRs/MAs) registration was the least-endorsed requirement (*n* = 9, 4.8%). Most journals that endorsed reporting guidelines and study registration provided a corresponding website for further reference.

### Multivariable linear regression analysis

Residual analysis showed that multivariable linear models basically conformed to linearity, normality, and homoscedasticity. The collinearity of 42 multivariable linear models was weak and had little influence on the stability of the results (VIF: 1.061–2.116). The start year, language, region, publisher, and journal size (only used to adjust total citations) were adjusted and showed that a journal’s IF was associated with the endorsement of RCT registration (geometric means ratio (GR) = 1.422, 95% CI [1.197–1.694]), CONSORT statement (GR = 1.318, 95% CI [1.104–1.578]), PRISMA statement (GR = 1.390, 95% CI [1.148–1.683]), STROBE statement (GR = 1.556, 95% CI [1.262–1.919]), STARD statement (GR = 1.585, 95% CI [1.216–2.070]), and MOOSE statement (GR = 2.113, 95% CI [1.422–3.133]) (Table 2). The endorsements of RCT registration (GR = 1.652, 95% CI [1.268–2.153]), CONSORT statement (GR = 1.570, 95% CI [1.199–2.061]), PRISMA statement (GR = 1.698, 95% CI [1.271–2.270]), STROBE statement (GR = 2.023, 95% CI [1.476–2.773]), STARD statement (GR = 2.173, 95% CI [1.452–3.243]), and MOOSE statement (GR = 2.249, 95% CI [1.219–4.150]) were associated with the total number of citations (Table 3). The univariable analysis and multivariable analysis results were consistent.

**Table 2:** The association between the endorsement of reporting guidelines or study registration and Journal Impact Factor (2018) in Surgery journals.

Requirements	Number of endorsed journals (%)	Univariable analysis		Multivariable linear regression^a^	
		GR (95% CI)^b^	*P* value	GR (95% CI)^b^	*P* value
Disclosure of COI	170 (90.4)	0.865 [0.617–1.211]	0.395	0.836 [0.612–1.140]	0.253
ICMJE recommendations	155 (82.4)	1.002 [0.771–1.303]	0.989	1.054 [0.839–1.327]	0.647
RCTs registration	101 (53.7)	1.406 [1.159–1.706]	0.001*	1.422 [1.197–1.694]	<0.001*
CONSORT statement	94 (50.0)	1.321 [1.089–1.607]	0.005*	1.318 [1.104–1.578]	0.003*
PRISMA statement	66 (35.1)	1.384 [1.127–1.694]	0.002*	1.390 [1.148–1.683]	0.001*
EQUATOR network	56 (29.8)	1.205 [0.971–1.496]	0.091	1.242 [0.998–1.542]	0.052
STROBE statement	51 (27.1)	1.581 [1.276–1.959]	<0.001*	1.556 [1.262–1.919]	<0.001*
ARRIVE statement	44 (23.4)	0.942 [0.745–1.191]	0.621	0.968 [0.774–1.211]	0.778
STARD statement	35 (18.6)	1.390 [1.081–1.786]	0.011*	1.585 [1.216–2.070]	0.001*
CARE statement	27 (14.4)	0.851 [0.641–1.130]	0.262	0.933 [0.701–1.242]	0.631
COREQ statement	21 (11.2)	1.279 [0.935–1.754]	0.123	1.309 [0.942–1.820]	0.108
CHEERS statement	21 (11.2)	1.189 [0.867–1.629]	0.282	1.250 [0.902–1.734]	0.180
SPIRIT statement	20 (10.6)	1.081 [0.783–1.493]	0.634	1.052 [0.746–1.479]	0.775
PRISMA-P statement	18 (9.6)	1.016 [0.723–1.426]	0.931	1.002 [0.692–1.445]	1.000
TRIPOD statement	18 (9.6)	1.112 [1.426–1.560]	0.537	1.164 [0.805–1.679]	0.419
SQUIRE statement	16 (8.5)	1.005 [0.703–1.435]	0.977	1.042 [0.697–1.556]	0.843
SRQR statement	14 (7.4)	1.028 [0.703–1.503]	0.887	1.151 [0.728–1.820]	0.547
RIGHT statement	12 (6.4)	0.847 [0.564–1.271]	0.421	0.805 [0.514–1.259]	0.340
AGREE statement	12 (6.4)	0.847 [0.564–1.271]	0.421	0.805 [0.514–1.259]	0.340
MOOSE statement	9 (4.8)	2.301 [1.466–3.614]	<0.001*	2.113 [1.422–3.133]	<0.001*
SRs/MAs registration	9 (4.8)	1.211 [0.759–1.928]	0.421	1.091 [0.724–1.641]	0.676

**Notes:**

* The difference was statistically significant.

a The covariables that need to be adjusted in multivariable linear regression are start year, language, region, publisher.

b GR (95% CI) : geometric means ratio (95% confidence interval), this value is calculated by using the “not endorsed” group as the reference group. For example, GR = 1.422 says that the value of Impact Factor is 42.2% higher for journals that endorse RCT registration, the same is true for other results.

**Table 3:** The association between the endorsement of reporting guidelines or study registration and Journal Total Cites (2018) in Surgery journals.

Requirements	Number of endorsed journals (%)	Univariable analysis		Multivariable linear regression^a^	
		GR (95% CI)^b^	*P* value	GR (95% CI)^b^	*P* value
Disclosure of COI	170 (90.4)	0.621 [0.325–1.183]	0.147	0.703 [0.440–2.831]	0.142
ICMJE recommendations	155 (82.4)	0.724 [0.439–1.194]	0.204	0.989 [0.706–1.445]	0.954
RCTs registration	101 (53.7)	1.489 [1.019–2.178]	0.040*	1.652 [1.268–2.153]	<0.001*
CONSORT statement	94 (50.0)	1.581 [1.086–2.307]	0.017*	1.570 [1.199–2.061]	0.001*
PRISMA statement	66 (35.1)	1.945 [1.318–2.871]	0.001*	1.698 [1.271–2.270]	<0.001*
EQUATOR network	56 (29.8)	1.230 [0.811–1.866]	0.329	1.300 [0.935–1.811]	0.119
STROBE statement	51 (27.1)	2.350 [1.556–3.548]	<0.001*	2.023 [1.476–2.773]	<0.001*
ARRIVE statement	44 (23.4)	1.030 [0.656–1.618]	0.895	1.009 [0.703–1.393]	0.953
STARD statement	35 (18.6)	1.910 [1.178–3.090]	0.009*	2.173 [1.452–3.243]	<0.001*
CARE statement	27 (14.4)	0.644 [0.375–1.107]	0.111	0.847 [0.550–1.309]	0.454
COREQ statement	21 (11.2)	1.560 [0.853–2.851]	0.148	1.545 [0.938–2.547]	0.088
CHEERS statement	21 (11.2)	1.202 [0.656–2.208]	0.549	1.279 [0.776–2.104]	0.332
SPIRIT statement	20 (10.6)	1.153 [0.621–2.143]	0.649	1.138 [0.676–1.910]	0.627
PRISMA-P statement	18 (9.6)	1.122 [0.586–2.148]	0.727	1.096 [0.627–1.914]	0.746
TRIPOD statement	18 (9.6)	1.219 [0.637–2.333]	0.548	1.294 [0.741–2.265]	0.362
SQUIRE statement	16 (8.5)	1.256 [0.634–2.489]	0.511	1.294[0.705–2.382]	0.403
SRQR statement	14 (7.4)	1.346 [0.652–2.786]	0.420	1.589 [0.794–3.177]	0.190
RIGHT statement	12 (6.4)	1.230 [0.562–2.685]	0.602	1.148 [0.581–2.275]	0.689
AGREE statement	12 (6.4)	1.230 [0.562–2.685]	0.602	1.148 [0.581–2.275]	0.689
MOOSE statement	9 (4.8)	3.304 [1.371–7.962]	0.008*	2.249 [1.219–4.150]	0.010*
SRs/MAs registration	9 (4.8)	0.946 [0.386–2.317]	0.903	0.940 [0.571–1.982]	0.843

**Notes:**

* The difference was statistically significant.

a The covariables that need to be adjusted in multivariable linear regression are start year, language, region, publisher and journal size.

b GR (95% CI): geometric means ratio (95% confidence interval), this value is calculated by using the “not endorsed” group as the reference group. For example, GR = 1.652 says that the number of citations is 65.2% higher for journals that endorse RCT registration, the same is true for other results.

## Discussion

We investigated the extent to which author instructions in surgical journals endorsed different reporting guidelines and study registration procedures. The relationship between these requirements and the journal’s IF or total citations was then analyzed. Our research data showed that COI disclosure was most likely to be endorsed (90.4%). About four-fifths (86.7%) of journals endorsed one or more reporting guidelines, and approximately half (54.8%) of the journals endorsed implementing study registration. Other similar studies found the endorsement of reporting guidelines and study registration in 52.2% (35/67) and 22.4% (15/67) of hematology journals (Wayant et al., 2017), 59.5% (22/37) and 43.2% (16/37) of critical care journals (Sims et al., 2018), and 59.3% (16/27) and 44.4% (12/27) of emergency medicine journals (Sims et al., 2016). Interestingly, the order of degrees, rather than the proportion, of endorsement found in surgical journals were very similar to those of journals in other medical fields (Hopewell et al., 2008; Knüppel et al., 2013; Kunath et al., 2011; Kunath et al., 2012; Meerpohl et al., 2010). Some requirements (such as COI disclosure, ICMJE recommendations, RCT registration, CONSORT statement, and PRISMA statement) have higher rates of compliance, perhaps because researchers are more familiar with them. None of the other requirements were endorsed by more than 30% of journals.

We also found that higher IF was associated with the endorsement of a “CONSORT statement” (GR = 1.318, 95% CI [1.104–1.578]), “PRISMA statement” (GR = 1.390, 95% CI [1.148–1.683]), “STROBE statement” (GR = 1.556, 95% CI [1.262–1.919]), “STARD statement” (GR = 1.585, 95% CI [1.216–2.070]), “MOOSE statement” (GR = 2.113, 95% CI [1.422–3.133]), or “RCT registration” (GR = 1.422, 95% CI [1.197–1.694]) in surgical journals. The endorsement of a “CONSORT statement” (GR = 1.570, 95% CI [1.199–2.061]), “PRISMA statement” (GR = 1.698, 95% CI [1.271–2.270]), “STROBE statement” (GR = 2.023, 95% CI [1.476–2.773]), “STARD statement” (GR = 2.173, 95% CI [1.452–3.243]), “MOOSE statement” (GR = 2.249, 95% CI [1.219–4.150]), or “RCT registration” (GR = 1.652, 95% CI [1.268–2.153]) showed an association with a greater number of total citations too. Journals that endorsed these requirements were more likely to correspond to higher IF and more total citations, perhaps because strict manuscript submission standards improved the quality of the publication to some extent. Additionally, empirical studies have demonstrated a real improvement in research quality after the introduction of reporting guidelines and a study registration mechanism (Altman et al., 2008; Limb et al., 2019; Matheson, 2011; Moher et al., 2008; Smith et al., 2015).

Reputable journals are widely read because of their influence on medical practices, and the influence of journals is traditionally measured using IF and total citations (Jones, Huggett & Kamalski, 2011; Trueger et al., 2015). Authors are primarily responsible for the quality of their manuscripts and should completely and accurately report their findings. However, many authors do not have the ability or experience to do so (Kunath et al., 2012). Esene et al. (2018) found that research design was frequently misrepresented in neurosurgical literature and that mislabeling research impairs the indexing, classification, and sorting of evidence. Journals that continue to accept manuscripts with substandard reporting provide little incentive for authors to meet the higher standards outlined by their reporting guidelines (Camm, Agha & Edison, 2015). A journal’s submission guidelines are a basic threshold for a paper’s publication. As the “gatekeeper” of scientific research, journal publishers play a vital role in controlling the quality of published papers. Surgical journal policies may help improve article quality by forcing compliance with reporting guidelines and the registration of clinical trials. We recommend that journals endorse reporting guidelines and study registration and take steps to promote the adequate reporting of methods and results in accordance with these guidelines. We also recommend that trial registration numbers are submitted in manuscripts. Journals can ensure that each paper meets the minimum standards for publication by implementing these policies and increasing submission requirements, while simultaneously providing the public with more comprehensive trial information and increasing confidence in their results.

Though our study did include a variety of reporting guidelines, there were some limitations to our study that might have impacted our results. We did not collect requirements that were only made known during the submission process and our results may differ slightly from journal editorial policies. Additionally, we did not know how various requirements were fulfilled, which means the journal’s endorsement rate for reporting guidelines and study registration may be overestimated. Our statistical analyses considered some confounding factors that may have influenced the results and other confounding factors (such as number of reviewers, review cycle, and whether statisticians are involved) may not have been included in this study. Future studies should try to collect as many of these factors as possible to better correct for the confounders, which may require researchers to get in touch with journal editors *via* email to gather information that is not publicly available. Additionally, since we surveyed only the two most common types of study registrations (RCT and Systematic Reviews/Meta-analyses), our study is not comprehensive. Some journals do not publish certain types of research and some reporting guidelines did not apply, which may have led to an inaccurately lower rate of endorsement for the corresponding reporting guidelines. In order to minimize this bias, researchers can try to contact the editor to determine what types of articles the journal does not publish. Lastly, the cross-sectional nature of the study means that temporality could not be assessed, and an explanation that higher quality journals were more likely to endorse reporting guidelines could not be excluded.

## Conclusion

In conclusion, the endorsement of reporting guidelines and study registration was associated with higher IF and more total citations in surgical journals. Good publishing practices are critical for the long-term development of journals and the improvement of their reputations. Therefore, we encourage journals and publishers to follow these requirements to strengthen the value of health research and journals themselves as much as possible. Additional studies are needed to obtain empirical data on the relationship between reporting guidelines or study registration and the quality of journals in other fields of medicine.

## Supplemental Information

10.7717/peerj.12837/supp-1 Supplemental Information 1 The data of raw measurements of the included journals. All requirements are divided into “endorsed group” and not endorsed group and used to explore the requirements that may be associated with the Journal Impact Factor and Total Cites.

10.7717/peerj.12837/supp-2 Supplemental Information 2 The information on the journals that were excluded from this study.

10.7717/peerj.12837/supp-3 Supplemental Information 3 The distribution of the Impact Factor 2018 in sample surgery journals (*n* = 188).

10.7717/peerj.12837/supp-4 Supplemental Information 4 The distribution of the Total Cites 2018 in sample surgery journals (*n* = 188).

## Additional Information and Declarations

### Data Availability

The following information was supplied regarding data availability: The raw measurements and excluded data are available in the Supplemental Files.
