# Supplementary figures and images for: The relationship between endorsing reporting guidelines or trial registration and the impact factor or total citations in surgical journals
Source: PeerJ. 2022 Jan 25;10:e12837. doi: 10.7717/peerj.12837 (PMC8796708; doi:10.7717/peerj.12837)

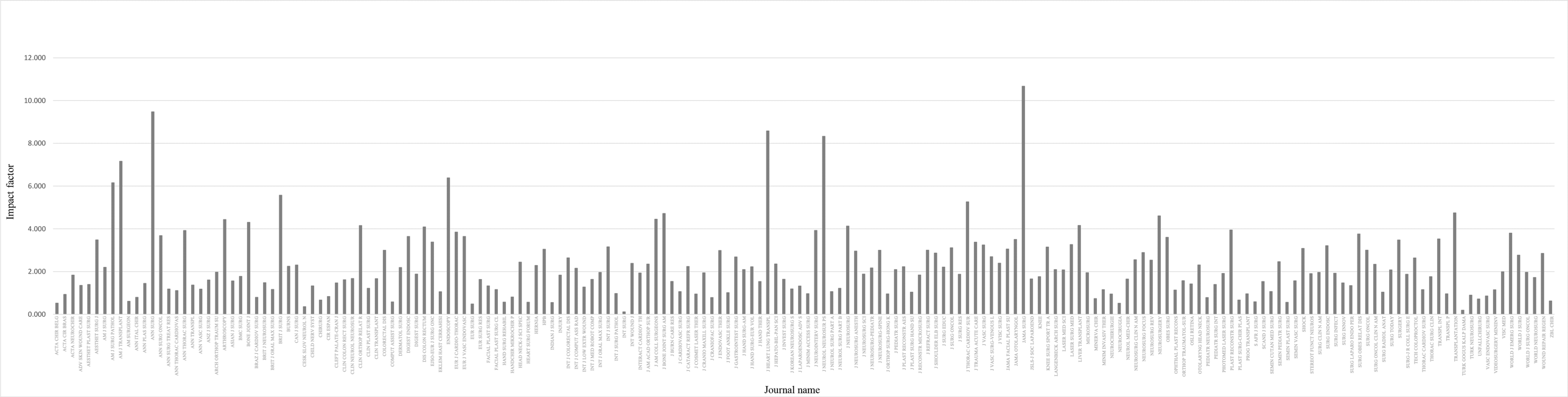

Supplement: Supplemental Information 3 [file peerj-10-12837-s003.png]

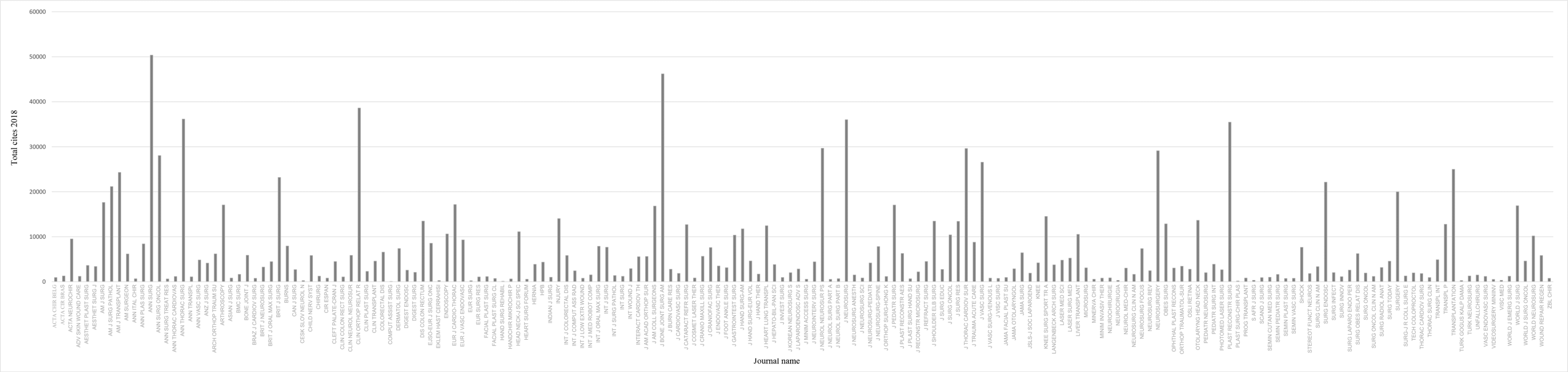

Supplement: Supplemental Information 4 [file peerj-10-12837-s004.png]
